# The feasibility of combining low-level exercise with vasodilator stress in patients referred for stress perfusion cardiac MRI

**DOI:** 10.1186/1532-429X-18-S1-Q12

**Published:** 2016-01-27

**Authors:** Jason Craft, Debbie Scandling, Orlando P Simonetti, Beth McCarthy, Vikram Brahmanandam, Sharath Subramanian, Juliana Serafim da Silveira, Subha V Raman

**Affiliations:** 1 Cardiology, Advocate Christ Medical Center, Oak Lawn, IL USA; 2 Cardiology, Ohio State University, Columbus, OH USA

## Background

Adenosine, an agonist of the A2a receptor, is widely used for stress CMR. However, target receptors for adenosine are heterogeneous in their location and facilitated physiologic effects. A2b and A3 receptors are responsible for bronchospasm and peripheral arteriolar vasodilation; A1 receptors are responsible for AV block.

The rate of adverse reactions with adenosine approaches 80%, consisting of dyspnea, headache, flushing, chest/abdominal discomfort, angina, ST depression, dizziness, nausea, and dysgeusia. In SPECT protocols, use of vasodilators has been previously established as safe during low level exercise, and results in fewer adverse reactions. Our hypothesis is that adenosine administered during low level exercise treadmill stress cardiac MRI is safe, feasible, and results in diagnostic quality imaging.

## Methods

For the purpose of this study, a MRI compatible treadmill was used as previously described (Foster et al). 45 consecutive patients referred for clinically indicated stress CMR from 3/2014 through 4/2015 were eligible for enrollment. After informed consent, patients were prospectively randomized to undergo either vasodilator stress CMR (VS CMR) or low level exercise vasodilator stress CMR (EVS CMR). In all, we attempted VS CMR on 11 patients, and EVS CMR on 10 patients. 21 patients were excluded due to pre-existing orthopedic/neurologic conditions, or patient preference.

**Figure 1:**
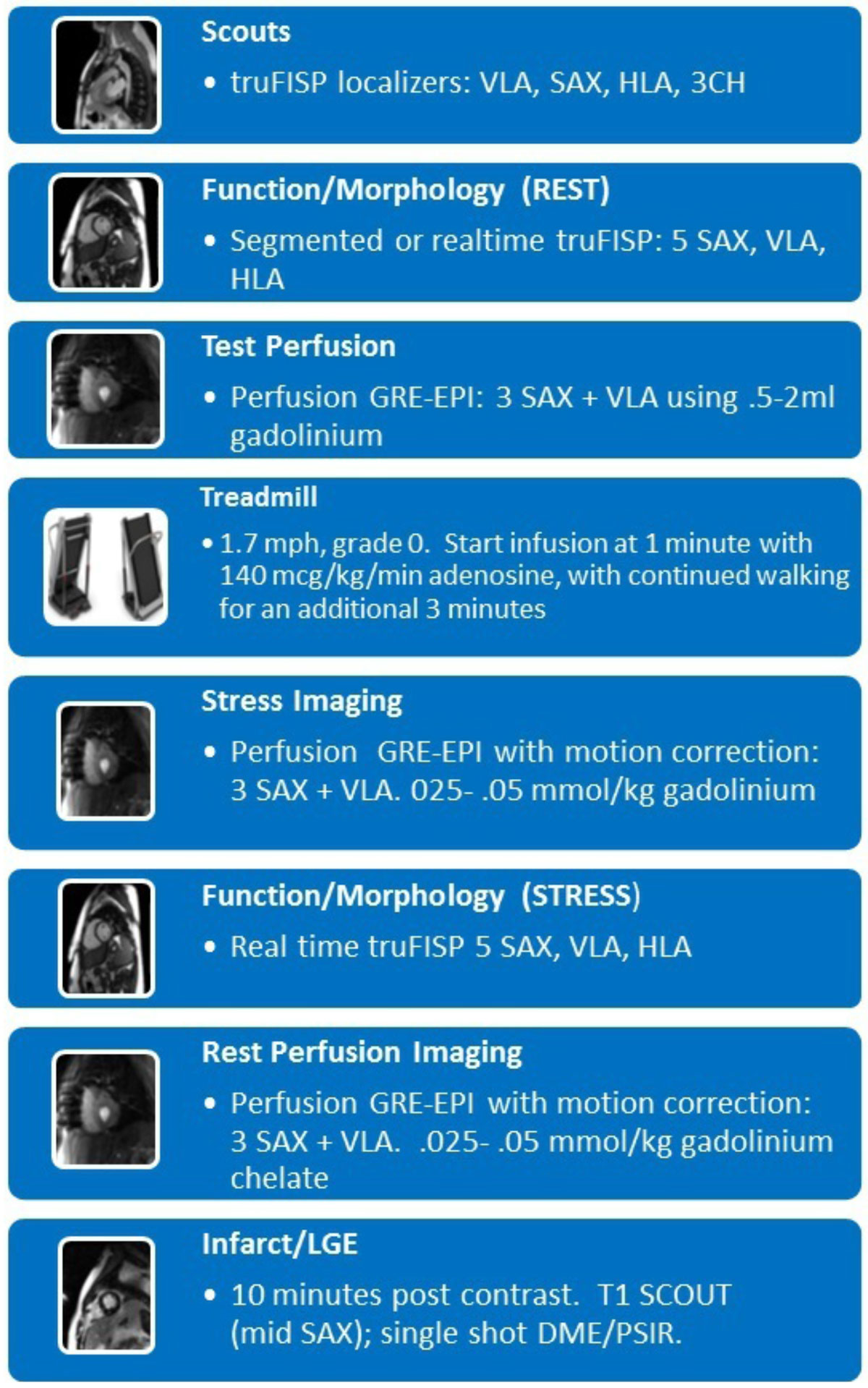
**The EVS CMR protocol**.

The EVS CMR protocol consisted of a 1.7 mph, 0% grade treadmill walk for a total of 4 minutes; with staggered infusion of adenosine 140 mcg/kg/min starting after one minute of exercise. Adenosine administration continued for an additional minute post exercise. Imaging commenced at approximately 30 seconds after exercise termination and injection of gadolinium. Symptoms in both groups were recorded. Monitoring parameter included continuous EKG, HR, blood pressure, and pulse oximetry. Image fidelity was evaluated upon completion of the final report according to a 4-point scale (4= best, 1=worst). All imaging was performed on a Siemens Avanto 1.5 T system. The imaging protocol is outlined in figure 1.

## Results

A comparison between both groups is found in Table 1. 9 patients successfully completed the EVS CMR protocol, with one test terminated due to bronchospasm. 10 patients successfully completed the VS CMR protocol, with one test terminated due to loss of IV access. In the EVS CMR group, there were no adverse cardiovascular or mechanical safety events. The average image score of the EVS CMR group was 3.57 +/- .53, versus 3.60 +/- .51 (VS CMR) p=.913. All studies were of diagnostic value. The average transition time (the time elapsed from the conclusion of exercise to the beginning of imaging) in EVS CMR patients was 30 seconds.

**Table 1:** A Comparison of EVS CMR vs VS CMR Groups

Group	EVS CMR (treadmill)	VS CMR (adenosine only)
	N = 9	N = 10
Age	63.1 +/- 10.6	54.5 +/- 12.6
Image Quality Score	3.57 +/- .53*	3.60 +/- .51
Ischemia Present	2	3
LGE Present (total)	5	7
_midwall/RV insertion_	2	4
_subendocardial/transmural_	3	1
_epicardial_	0	2
Known CAD	8	3
Transition time	30.01 sec	N/A
Indication for CMR		
_CAD/MI_	3	3
_chest pain_	4	4
_arrhythmia_	1	3
_cardiomyopathy_	1	2
_abnormal EKG_	1	0
_dyspnea_	5	2

*p = .913

## Conclusions

EVS CMR is safe, feasible, and results in diagnostic quality imaging. Further analysis is needed to determine the effect of EVS CMR on the incidence of adenosine induced adverse reactions.

